# Draft Genome Sequence of *Pseudoalteromonas luteoviolacea* Strain B (ATCC 29581)

**DOI:** 10.1128/genomeA.00048-13

**Published:** 2013-02-28

**Authors:** Brady F. Cress, Kelly A. Erkert, Blanca Barquera, Mattheos A. G. Koffas

**Affiliations:** a Department of Chemical and Biological Engineering, Center for Biotechnology and Interdisciplinary Studies, Rensselaer Polytechnic Institute, Troy, New York, USA; b Department of Biology, Center for Biotechnology and Interdisciplinary Studies, Rensselaer Polytechnic Institute, Troy, New York, USA

## Abstract

We report the 4.049-Mbp high-quality draft assembly of the *Pseudoalteromonas luteoviolacea* strain B (ATCC 29581) genome. This marine species is known to biosynthesize several antimicrobial compounds, including the purple pigment violacein. Whole-genome sequencing and genome mining will complement experimental studies aimed at elucidating novel biosynthetic pathways capable of producing pharmaceutically relevant molecules. Based upon 16S rRNA phylogenetic analysis, we propose that strain ATCC 29581 be classified as a distinct phylogenetic species of the genus *Pseudoalteromonas*.

## GENOME ANNOUNCEMENT

Strain ATCC 29581 (*Pseudoalteromonas luteoviolacea* B) is a marine bacterium that was isolated from surface seawater in Kinko Bay at the extreme southern end of the Japanese island Kyushu (1). Originally belonging to the genus *Alteromonas*, the species was reclassified as *Pseudoalteromonas luteoviolacea* after small-subunit rRNA phylogenetic analysis of several *Alteromonas* species revealed evolutionarily divergent clusters appropriately split into two distinct genera, *Alteromonas* and *Pseudoalteromonas* (2). Initial phylogenetic analysis (data not shown) of the 16S rRNA sequence from strain ATCC 29581 against a representative group of pseudoalteromonads suggests that this organism is more closely related to *Pseudoalteromonas ulvae*, although it exists on a divergent phylogenetic branch from all type species. Given this evidence, we propose that strain ATCC 29581 was misclassified as *Pseudoalteromonas luteoviolacea* based on phenotypic analysis and should instead be classified as a distinct, new phylogenetic species in the genus *Pseudoalteromonas*.

Genomic DNA was purified from strain ATCC 29581 utilizing the Wizard genomic DNA purification kit (Promega). The genome was sequenced using the Illumina HiSeq 2000 sequencing system, which produced 68 M paired-end reads of 101 bp with an insert size of 400 bp. Approximately 29 M random reads were assembled with Velvet v1.2.07 (3) at an optimal hash length of 95. The final genome assembly has 41-fold coverage and contains 49 supercontigs composed of 61 contigs (>200 bp in length) with a total size of 4,048,690 bp, an N_50_ contig length of 375,446 nucleotides, and a mean G+C content of 41.9%. All assembly data were deposited in the EMBL nucleotide sequence database.

The draft genome was annotated by the RAST (Rapid Annotation using Subsystem Technology) server (4) using Glimmer3 as a gene caller (5), which predicted 3,681 coding sequences (CDSs) with an average length of 980 bp (2,396 CDSs have functional predictions), 99 tRNA-encoding genes, and 7 rRNA-encoding genes. RAST was also used to construct a draft metabolic model (6) containing 718 genes, corresponding to 1,053 reactions with 958 metabolites (including 4 gap-filling reactions and an artificial biomass reaction). Further genome analysis will help uncover biosynthetic pathways and enzymes capable of performing unique and valuable biotransformations.

### Nucleotide sequence accession numbers.

The annotated draft genome sequence was deposited in DDBJ/EMBL/GenBank under accession no. CAPN00000000. The version described in this paper is the first version, CAPN01000000.
